# ﻿New records of *Provanna* (Gastropoda, Provannidae) from the Costa Rica Margin and an identification key for the genus

**DOI:** 10.3897/zookeys.1189.109734

**Published:** 2024-01-12

**Authors:** Melissa J. Betters, Erik E. Cordes

**Affiliations:** 1 Department of Biology, Temple University, Philadelphia, PA, USA Temple University Philadelphia United States of America

**Keywords:** Gastropoda, identification, species delimitation, systematics, taxonomy

## Abstract

Consistent species identification is foundational to biological research and requires coordination among a diversity of researchers and institutions. However, such consistency may be hindered for rare organisms where specimens, identification resources, and taxonomic experts are few. This is often the case for deep-sea taxonomic groups. For example, the deep-sea gastropod genus *Provanna* Dall, 1918 is common at chemosynthetic sites throughout the world’s oceans, yet no formal guide to these species has yet been produced. Recent exploration has recovered new specimens of *Provanna* from hydrocarbon seeps off the Pacific Costa Rica Margin. The current work assesses the species identity of these specimens using shell morphology, radular morphology, and genetic barcoding (mitochondrial CO1 and nuclear H3). Records of occurrence for *P.laevis* Warén & Ponder, 1991, *P.ios* Warén & Bouchet, 1986, and *P.pacifica* Warén & Bouchet, 1986 are herein presented from the Costa Rica Margin. A critical taxonomic review of the 29 extant species within this genus was conducted and their genetic, morphological, and biogeographical distinction assessed. In this review, genetic and morphological support was found for nearly all current species delineations except for *P.glabra*Okutani et al., 1992, **syn. nov.** and *P.laevis*, **syn. nov.**, which are herein synonymized to *P.laevis*, and for *P.ios*, **syn. nov.** and *P.goniata* Warén & Bouchet, 1986, **syn. nov.**, which are synonymized to *P.ios*. Finally, the first species identification key for the extant species in this genus is presented. This work clarifies the taxonomy and systematics of this deep-sea gastropod genus and contributes a novel polytomous key for use in future research.

## ﻿Introduction

Consistent species identification is foundational to biological research. Studies of populations, communities, and ecosystems all rely on authors from a wide range of backgrounds and locations coordinating species definitions. This is particularly salient for the global endeavor of deep-sea research, where many species are recent discoveries. As many regions of the ocean remain unexplored, taxonomic characterization of marine invertebrates are hindered by a lack of collections, occurrence records, identification resources, and taxonomic experts (Sigwart et al. 2019; Engel et al. 2021).

Throughout the world’s oceans, ecosystems reliant on chemosynthetic activity, such as hydrothermal vents and hydrocarbon seeps, are hotspots for productivity on the ocean floor, hosting an anomalously high biomass community consisting of numerous endemic species (Sibuet and Olu 1998). These ecosystems often host abundant gastropod populations which act as the primary grazers at these sites, feeding on the biofilms and bacterial mats that coat the hard surfaces of these environments (Sasaki et al. 2010). To identify such gastropods to the species level, researchers commonly rely on data sources such as shell morphology, genetic barcoding (e.g., mitochondrial cytochrome oxidase I gene (CO1)), and radular imaging (e.g., Nekhaev 2023). However, not all known species have been genetically barcoded, precluding the utility of these data in every case. With few taxonomic resources available for these ecosystems, researchers must rely on formal descriptions to identify species which assume a thorough, prior knowledge of taxon-specific language. Furthermore, comparisons across dozens of species may be necessary to come to a confident identification which can be arduous and time-consuming, particularly when species identification is just one part of a broader study. A solution to this problem is a key, which synthesizes a set of informative criteria and guides researchers to a workable species hypothesis. Keys are typically more easily implemented by researchers across expertise levels than formal descriptions, thus presenting an effective method with which to streamline and standardize species identifications.

The current work centers on aa genus of Abyssochrysoid snails *Provanna* (Dall, 1918), presenting new records, a taxonomic review, and a new identification key for its species. *Provanna* occur worldwide and are endemic to chemosynthetic environments (Johnson et al. 2010; Amano and Little 2012; Linse et al. 2019). In total, 29 extant species of *Provanna* are currently recognized on the World Register of Marine Species (WoRMs), six of which have recently been designated as Endangered or Critically Endangered on the IUCN Red List (Molloy et al. 2020a, b, c; Thomas and Sigwart 2020; Molloy and Thomas 2021a, b; IUCN 2022). Despite their cosmopolitan distribution and their threatened status, however, identification resources for this group are lacking, increasing the likelihood that some of the recognized species are synonymous and that other species are cryptic and have gone unrecognized within this genus. An understanding of the conservation status of species must begin with a confident identification of the species.

Species of *Provanna*, like other deep-sea gastropods, may be distinguished by their shell and radular morphology, making a morphology-based identification key useful. All *Provanna* share certain characteristics that distinguish the genus. Specimens have small, turbinate, dextral shells, a thin periostracum, no umbilicus, and usually no more than 2–3 shell whorls intact, regardless of size. They are never wider than they are tall and their apertures have a distinct shape; They are rarely circular or ovate. Rather, the columellar lip typically curves inwards near the bottom of the shell, such that it forms a near-right angle with the lower lip (see Fig. 2A for an example). They are also small, with one of the largest specimens recorded just 2 cm in length (Chen et al. 2018). They may be distinguished from their sister genus *Desbruyeresia* in that *Desbruyeresia* typically have tall, intact spires of > 3–4 whorls and more slender, turriform shells (Warén and Bouchet 1993). They also have distinct radular characteristics, with *Desbruyeresia* having multiple denticles on the cusps of their central teeth while *Provanna* have none (Warén and Bouchet 1993). Protoconchs (larval shells) are also useful in distinguishing these genera; However, protoconchs are almost unanimously missing in *Provanna* specimens (Warén and Ponder 1991; Warén and Bouchet 1993). Finally, genetic characterization of this group is still ongoing, with gene sequences currently unavailable for *P.abyssalis* (Okutani & Fujikura, 2002), *P.admetoides* (Warén & Ponder, 1991), *P.chevalieri* (Warén & Bouchet, 2009), *P.goniata* (Warén & Bouchet, 1986), *P.muricata* (Warén & Bouchet, 1986), *P.nassariaeformis* (Okutani, 1990), nor *P.reticulata* (Warén & Bouchet, 2009). This lack of sequence data makes an identification key based on morphology particularly relevant, especially one that is created using an integrative taxonomy approach that includes genetic data.

In the present study, we present formal records of *Provanna* from hydrocarbon seeps at the Costa Rica Margin that were sampled from 700 to 2000 meters depth. These sites were sampled during three cruises from 2017–2019, representing one of the most intensive sampling efforts in this region to date. The hydrocarbon seepage in this region is driven by the subduction of the Cocos Plate beneath the Caribbean plate (Suess 2014) and fuels chemosynthetic primary productivity at these sites (Cavanaugh et al. 1981; Suess 2014). This investigation aims to identify these Costa Rican hydrocarbon seep *Provanna* down to the species level using both morphological and genetic data. While the ecology and depth partitioning of these *Provanna* specimens have been recently investigated (Betters et al. 2023), the present study details the rigorous taxonomic identification of these specimens. We incorporate these data into a holistic review of the genus with the aim of assessing the morphological and genetic distinction among its extant species. Finally, we synthesize these results into the first polytomous species identification key for all currently known, extant *Provanna* species.

## ﻿Materials and methods

### ﻿Specimen collection

**Figure 1.**
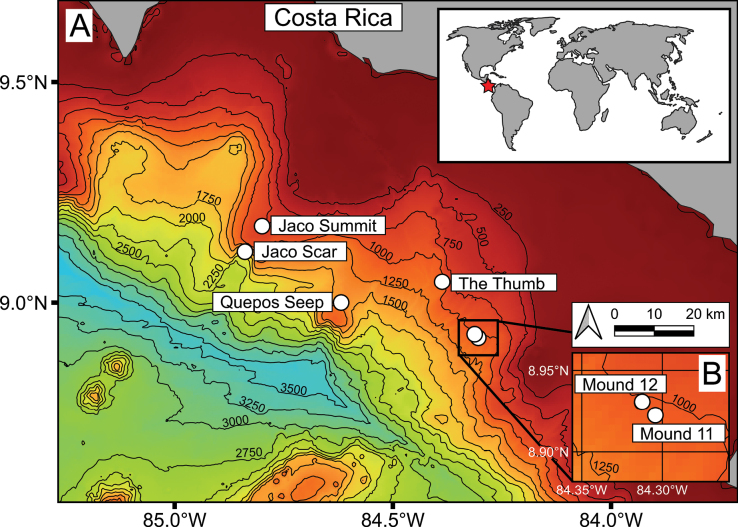
Map of the Costa Rica Margin **A** overview of the Costa Rica continental shelf with hydrocarbon seep sites labelled **B** close-up view of the sites Mound 12 and Mound 11, which appear overlapping in the larger map. Bottom bathymetry is demarcated by black lines every 250 m.

Specimens of *Provanna* were obtained from one of six sites at the Pacific Costa Rica Margin (CRM) during research expeditions conducted from 2017 to 2019 (Fig. 1, Table 1). *Provanna* were sampled by the human-operated vehicle (HOV) ‘Alvin’ and the remotely operated vehicle (ROV) ‘Subastian’ using various sampling tools attached to the HOV or ROV such as the manipulator arm or suction hose. The locations of sampling events were recorded for all specimens collected. Upon arrival to the surface, specimens were kept cold before being promptly preserved in > 95% ethanol. Specimens were then stored long term in ethanol at room temperature (20–25 °C).

**Table 1.** Summary of hydrocarbon seep sites sampled at the Costa Rica Margin and their associated species yield. Abbreviations in cruise identities are defined as follows: FK = R/V ‘Falkor’ cruise number, AT = R/V ‘Atlantis’ cruise number. Abbreviations: SD = ROV ‘Subastian’ dive number, AD = HOV ‘Alvin’ dive number. *Previously identified as *P.goniata*.

Seep site	Number of specimens	Species composition	GPS coordinates	Depth (m)	Cruise ID	Dive ID
Jaco Summit	6	*P.laevis* (100%)	9.174°N, 84.800°W	740–760	FK19-0106 AT37-10 AT37-13	SD213 AD4874 AD4912 AD4913 AD4914
Jaco Scar	180	*P.ios* (100%)*	9.115°N, 84.836°W	1800–2000	AT42-03	AD4971 AD4973 AD4977 AD4989
					FK19-0106	SD214
Quepos Seep	6	*P.pacifica* (100%)	8.922°N, 84.305°W	1000–1100	AT37-13	AD4924
The Thumb	815	*P.laevis* (100%)	9.049°N, 84.354°W	1071–1075	FK19-0106	SD217
Mound 12	803	*P.laevis* (100%)	8.930°N, 84.313°W	900–1050	AT37-13	AD4907 AD4910 AD4917
					AT42-03	AD4974 AD4978 AD4984 AD4985 AD4987
Mound 11	5	*P.lomana* (40%), *P.pacifica* (60%)	9.031°N, 84.619°W	1300–1500	AT42-03	AD4988

### ﻿Morphological analysis

**Figure 2.**
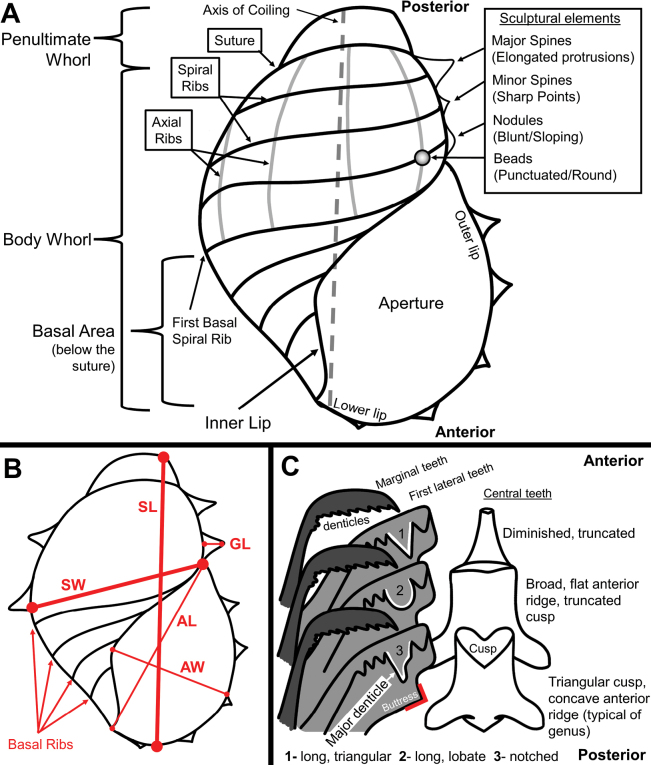
Visual definitions of morphological characters and measurements used in the study **A** shell morphological characters **B** informative measurements assessed for our own specimens **C** radular terminology and morphological characters. Abbreviations: SW: Shell Width, SL: Truncated Shell Length, GL: Maximum Granule Length, AL: Aperture Length, AW: Aperture Width.

All morphological characters and measurements are defined in Fig. 2 and Table 2. To begin identifying our specimens and constructing the key, original taxonomic descriptions of all extant *Provanna* species were obtained. The following shell characters were annotated for each species: The number of axial and spiral ribs on the body whorl, the relative strength of the body whorl sculptures, sculptural elements formed, how far down the body whorl the axial ribs extend, the presence of basal ribs, the average roundness (width / length) of the original holotype and paratype shells, the depth of the shell suture, and the descriptive shape of the aperture (Table 3). The following radular characters were also annotated: The relative width of the central teeth, the descriptive shape of the central teeth cusps, the roundedness of the central teeth’s anterior ridges, the number of denticles on the first lateral teeth, which denticle on the first lateral teeth is the most longest (“major” denticle), the descriptive shape of the first lateral major denticles, the angle of the first lateral posterior buttress, and the number of marginal tooth denticles (Table 3).

**Table 2.** Definitions for selected terminology used to describe morphological characters.

**Aperture**	The opening of the shell from which the gastropod body would protrude
**Axis of coiling**	The imaginary line that runs from the top of a shell’s spire to the tip of its base around which the shell is coiled
**Axial sculpture**	The sculpturing of the shell running parallel to the axis of coiling
**Growth lines**	Fine transverse lines marking shell growth. They are distinguished from axial sculpturing in that they are not raised or grooved.
**Sculpture**	Three-dimensional, linear ornamentation on the outer surface of the shell. These rise away from the shell surface
**Sculptural element**	Knob-, bead-, or spike-like protrusions that occur at intersections of the axial and spiral sculptures and that are raised higher than either sculpture. **Note**: If a Provanna shell has structural elements, it will typically have both an axial and spiral sculpture.
**Body whorl margin**	The length between the posterior end of the aperture and the previous suture line
**Spiral sculpture**	The sculpturing of the shell running perpendicular to the axis of coiling
**Suture**	Where the whorls of the shell are fused, including where the aperture is fused with the body whorl
**Whorl**	One complete revolution of shell growth

**Table 3.** Summary of morphological characteristics for the type specimens of each *Provanna* species.

**Species**	**Axial ribs on body whorl**	**Spiral ribs on body whorl**	**Relative strength of ribs on body whorl**	**Sculptural elements**		**Axial body sculpture extends to**…
*P.abyssalis* Okutani & Fujikura, 2002	0	0	NA	Absent		NA
*P.admetoides* Warén & Ponder, 1991	35–45	2–3	Variable	Minor spines/Absent		Posterior end of aperture
*P.annae* Nekhaev, 2023	0	0	NA	Absent		NA
*P.beebi* Linse et al., 2019	>20	0–6	Spiral > Axial	Beaded/ Absent		Anterior end of shell
*P.buccinoides* Warén & Bouchet, 1993	10–20	3–4	Spiral < Axial	Nodules		Posterior end of aperture
*P.chevalieri* Warén & Bouchet, 2009	10–20	0–3	Spiral < Axial	Absent		Mid-body whorl
*P.cingulata* Chen et al., 2018	0	4–6	Spiral > Axial	Absent		NA
*P.clathrata* Sasaki et al., 2016	10–20	3–5	Variable	Nodules/ Minor Spines		Posterior end of aperture
*P.cooki* Linse et al., 2019	0	0–5	Spiral > Axial	Absent		NA
*P.exquisita* Chen & Watanabe, 2022	14–18	2–3	Spiral > Axial	Major Spines/Keel		Posterior end of aperture
*P.fenestrata* Chen et al., 2019	16–20	1–2	Variable	Nodules/ Absent		Posterior end of aperture
*P.glabra* Okutani et al., 1992	0	0	NA	Absent		NA
*P.goniata* Warén & Bouchet, 1986	15–20	2–3	Spiral > Axial	Major Spines		Posterior end of aperture
*P.ios* Warén & Bouchet, 1986	15	2	Spiral > Axial	Minor Spines		Posterior end of aperture
*P.kuroshimensis* Sasaki et al., 2016	0	0	NA	Absent		NA
*P.laevis* Warén & Ponder, 1991	0	0	NA	Absent		NA
*P.lomana* Warén & Bouchet, 1986	10–20	0	Spiral < Axial	Absent		Posterior end of aperture
*P.lucida* Sasaki et al., 2016	0	0–3	Spiral > Axial	Absent		NA
*P.macleani* Warén & Bouchet, 1989	10–20	4–5	Spiral > Axial	Absent		Posterior end of aperture
*P.muricata* Warén & Bouchet, 1986	14–16	1–2	Spiral < Axial	Nodules/ Minor Spines		Posterior end of aperture
*P.nassariaeformis* Okutani, 1990	20–25	3–5	Spiral = Axial	Beaded		Anterior end of shell
*P.pacifica* Warén & Bouchet, 1986	12–16	2–3	Spiral > Axial	Nodules/ Minor Spines		Posterior end of aperture
*P.reticulata* Warén & Bouchet, 2009	0–15	2–4	Spiral = Axial	Minor Spines		Posterior end of aperture
*P.sculpta* Warén & Ponder, 1991	15	3	Spiral < Axial	Beaded		Posterior end of aperture
*P.segonzaci* Warén & Ponder, 1991	10–20	2–3	Spiral < Axial	Nodules/ Minor Spines		Posterior end of aperture
*P.shinkaiae* Okutani & Fujikura, 2002	10–20	2–3	Spiral > Axial	Major Spines		Posterior end of aperture
*P.stephanos* Chen et al., 2019	10–20	2–3	Spiral > Axial	Major Spines/Keel		Posterior end of aperture
*P.subglabra* Sasaki et al., 2016	0	0	NA	Absent		NA
*P.variabilis* Warén & Bouchet, 1986	0–20	1–3	Spiral > Axial	Nodules/ Absent		Posterior end of aperture
**Species**	**Basal spiral ribs**	**Width / Length**	**Depth of suture**	**Aperture shape description**	**Central tooth width**	**Central tooth cusp**
*P.abyssalis* Okutani & Fujikura, 2002	Absent	0.5	Constricted	Globose	Typical	Long, triangular
*P.admetoides* Warén & Ponder, 1991	Present	0.61	Moderate	Fusiform	Very narrow	Very truncated
*P.annae* Nekhaev, 2023	Absent	0.6	Constricted	Globose	Typical	Long, triangular
**Species**	**Basal spiral ribs**	**Width / Length**	**Depth of suture**	**Aperture shape description**	**Central tooth width**	**Central tooth cusp**
*P.beebi* Linse et al., 2019	Present	0.59	Moderate	Fusiform/ Semicircle	Broad	Blunt, truncated
*P.buccinoides* Warén & Bouchet, 1993	Present	0.63	Moderate	Globose	Broad	Blunt, truncated
*P.chevalieri* Warén & Bouchet, 2009	Present	0.55	Constricted	Globose	Broad	Very short, triangular
*P.cingulata* Chen et al., 2018	Absent	0.61	Constricted	Globose	Broad	Short, triangular
*P.clathrata* Sasaki et al., 2016	Present	0.61	Constricted	Fusiform	Typical	Long, triangular
*P.cooki* Linse et al., 2019	Absent	0.57	Constricted	Fusiform	Broad	Triangular
*P.exquisita* Chen & Watanabe, 2022	Present	0.55	Constricted	Semicircular	Typical	Triangular
*P.fenestrata* Chen et al., 2019	Present	0.59	Moderate	Variable	Typical	Triangular
*P.glabra* Okutani et al., 1992	Absent	0.6	Flat	Globose	Typical	Triangular, blunt
*P.goniata* Warén & Bouchet, 1986	Present	0.59	Moderate	Globose/ Fusiform	Typical	Long, triangular
*P.ios* Warén & Bouchet, 1986	Variable	0.54	Constricted	Fusiform/ Semicircle	NA	Long, triangular
*P.kuroshimensis* Sasaki et al., 2016	Absent	0.58	Flat	Fusiform	Typical	Long, triangular
*P.laevis* Warén & Ponder, 1991	Absent	0.56	Flat	Variable	Typical	Short, triangular
*P.lomana* Warén & Bouchet, 1986	Present	0.55	Moderate	Globose	Typical	Long, triangular
*P.lucida* Sasaki et al., 2016	Absent	0.59	Constricted	Globose	Typical	Short, triangular
*P.macleani* Warén & Bouchet, 1989	Present	0.61	Moderate	Fusiform	Very narrow	Very truncated
*P.muricata* Warén & Bouchet, 1986	Present	0.56	Constricted	Globose	Typical	Triangular
*P.nassariaeformis* Okutani, 1990	Present	0.7	Flat	Fusiform	Broad	Triangular, blunt, truncated
*P.pacifica* Warén & Bouchet, 1986	Present	0.61	Moderate	Fusiform	Very narrow	Very truncated
*P.reticulata* Warén & Bouchet, 2009	Present	0.57	Moderate	Fusiform	Broad	Blunt, truncated
*P.sculpta* Warén & Ponder, 1991	Present	0.55	Moderate	Fusiform	Typical	Long, triangular
*P.segonzaci* Warén & Ponder, 1991	Present	0.59	Constricted	Fusiform	Typical	Triangular
*P.shinkaiae* Okutani & Fujikura, 2002	Present	0.51	Moderate	Semicircle	Typical	Long, triangular
*P.stephanos* Chen et al., 2019	Present	0.61	Flat	Globose	Typical	Short, triangular
*P.subglabra* Sasaki et al., 2016	Absent	0.61	Flat	Fusiform	Typical	Long, triangular
*P.variabilis* Warén & Bouchet, 1986	Variable	0.55	Moderate	Globose	Typical	Long, triangular
**Species**	**Central tooth anterior ridge**	**First lateral tooth cusps**	**First lateral major cusp**	**First lateral major cusp shape**	**First lateral buttress angle**	**Marginal tooth cusps**
*P.abyssalis* Okutani & Fujikura, 2002	Concave	6–7	2^nd^	Triangular, fused with first	Right	9–10
*P.admetoides* Warén & Ponder, 1991	Rounded	3–4	2^nd^	Spatulate	Obtuse	Alternating 7 or 14+
*P.annae* Nekhaev, 2023	Concave	3–4	2^nd^	Long, lobate	Right	7–9
*P.beebi* Linse et al., 2019	Flat	4–5	2^nd^	Truncated, lobate	Absent	13–17
*P.buccinoides* Warén & Bouchet, 1993	Rounded/Flat	7	Fourth	Very truncated, lobate	Absent	~30
*P.chevalieri* Warén & Bouchet, 2009	Variable	4–5	2^nd^	Very truncated, lobate	Sloping/ Absent	13–18
*P.cingulata* Chen et al., 2018	Round/Flat	4–5	3^rd^ or 4^th^	Rhomboid	Absent	15–18
*P.clathrata* Sasaki et al., 2016	Concave	4–5	2^nd^	Long, triangular	Right/ Acute	9–10
*P.cooki* Linse et al., 2019	Concave	5–6	2^nd^	Long, lobate	Obtuse	11–14
**Species**	**Central tooth anterior ridge**	**First lateral tooth cusps**	**First lateral major cusp**	**First lateral major cusp shape**	**First lateral buttress angle**	**Marginal tooth cusps**
*P.exquisita* Chen & Watanabe, 2022	Concave	4–5	2^nd^	Truncated, lobate	Right/ Obtuse	20–24
*P.fenestrata* Chen et al., 2019	Concave	4–5	2^nd^	Long, triangular	Right	9–10
*P.glabra* Okutani et al., 1992	Concave	4–5	2^nd^	Long, lobate	Right/ Acute	8–12
*P.goniata* Warén & Bouchet, 1986	Concave	5–6	2^nd^	Long, triangular	Right/ Acute	15–25
*P.ios* Warén & Bouchet, 1986	Concave	4–5	2^nd^	Long, triangular	Right/ Acute	15–25
*P.kuroshimensis* Sasaki et al., 2016	Concave	4–5	2^nd^	Long, lobate	Right	10–13
*P.laevis* Warén & Ponder, 1991	Concave	4–5	2^nd^	Truncated, lobate	Acute	15–20
*P.lomana* Warén & Bouchet, 1986	Concave	4–5	2^nd^	Long, triangular	Acute	Alternating 7 or 14+
*P.lucida* Sasaki et al., 2016	Concave	4–5	2^nd^	Long, triangular	Right/ Acute	13–15
*P.macleani* Warén & Bouchet, 1989	Rounded	3–4	2^nd^	Spatulate	Sloping/ Absent	15–20
*P.muricata* Warén & Bouchet, 1986	Concave	4–5	2^nd^	Lobate	Sloping/ Obtuse	15–20
*P.nassariaeformis* Okutani, 1990	Concave/Flat	4–5	2^nd^	Truncated, lobate	Absent	15–20
*P.pacifica* Warén & Bouchet, 1986	Rounded	3	2^nd^	Lobate/Spatulate	Sloping/ Obtuse	5–7
*P.reticulata* Warén & Bouchet, 2009	Concave	2–3	2^nd^	Truncated, lobate	Absent	15–20
*P.sculpta* Warén & Ponder, 1991	Concave	3–5	2^nd^	Long, triangular	Acute	15–20
*P.segonzaci* Warén & Ponder, 1991	Concave	5–7	2^nd^	Multilobate	Right/ Obtuse	15–20
*P.shinkaiae* Okutani & Fujikura, 2002	Concave	2–5	2^nd^	Long, lobate	Acute	10–14
*P.stephanos* Chen et al., 2019	Concave	4–5	2^nd^ or 3^rd^	Long, triangular, blunt	Obtuse	12–14
*P.subglabra* Sasaki et al., 2016	Concave	4–5	2^nd^	Long, triangular	Right/ Obtuse	15–20
*P.variabilis* Warén & Bouchet, 1986	Concave	4–5	2^nd^	Long, triangular	Acute	Alternating 7 or 14+

To begin identifying the specimens from the CRM, they were first sorted into distinct morphotypes. Representatives from the full geographic, temporal, and size range of each morphotype were then selected for detailed morphological assessment. The following characters were measured for each specimen: Shell width (mm), truncated shell length (measured from the right, posterior tip of the penultimate whorl to the lowest point of the aperture (mm)), aperture length (mm), aperture width (mm), number of basal ribs (counted), relative shell texture (maximum granule length on the body whorl / truncated shell length), aperture roundness (width / length), and shell roundness (width / length). Truncated shell length was used as most *Provanna* lack any whorl past the penultimate whorl. Measurements of characters were taken from photographs captured by a mounted AmScope microscope adapter camera attached to a standard dissection microscope (Leica S6D, Leica Microsystems GmbH). A standardized 1-mm marker was present in every photo to allow for standardized measurements. Specimens were kept submerged in > 95% ethanol while images were taken. The line measurement tool within AmScope was used to measure morphological characters. To identify any potential collinearity among shell morphological characters, Pearson correlation coefficients (PCC) were calculated using the package Ggally (Schloerke et al. 2021) in R (v. 4.2.3; R Core Team 2022). One-way ANOVAs were then conducted in base R to identify significant differences in shell morphology among the morphotypes sampled from Costa Rica.

To characterize the radulae of each morphotype, we performed the following protocol. First, the body whorl of the shell was punctured using a sharp probe. The whole animal was then incubated in a 1.5 mL microcentrifuge tube containing a 10% solution of proteinase-k for 5–15 min at 56 °C. Incubation was monitored and terminated once tissue was visibly loose and degraded, but not fully digested. The microcentrifuge tube was removed from the heat source, pulse-vortexed three times, and then its contents were rinsed into a clean glass petri dish using deionized (DI) water. Under a dissection scope, the radular ribbon was identified, extricated from any remaining soft tissue, and moved to another clean glass petri dish containing DI water to further dilute the proteinase-k solution and prevent further breakdown of the radular ribbon. Silicon wafer chips cut into ~ 1 cm^3^ squares were used as mounting substrate for scanning electron microscopy. To mount the radula, a very small droplet of DI water was placed onto a chip. The radula was then placed into this water droplet and manipulated under a light microscope into a flat, teeth-up position using forceps or a sharp probe. Manipulation was most successful when the radula was wet but not submerged. The radula’s position was monitored and adjusted under a light microscope while the water was allowed to evaporate. Once dry, radulae naturally adhered to the chip’s surface and were then stored dry until imaging. Scanning electron microscopy was undertaken using a QuantaTM 450 FEG scanning electron microscope (FEI 2012) in its low-vacuum setting at Temple University College of Engineering’s Nano Instrumentation Center. High-quality images were obtained without sputter coating. Tentative morphological identities were then ascribed to our specimens.

### ﻿Genetic analysis

To confirm the morphological identifications, the cytochrome oxidase 1 (CO1) mitochondrial gene and the histone 3 (H3) nuclear gene were sequenced. Tissue was obtained by pulling aside the operculum and pinching off a small sample of tissue from the foot (approximately 1 mm^3^). This tissue was then digested and its DNA extracted using a Qiagen Blood and Tissue DNA Extraction kit (QIAGEN, Valencia, CA). Extracted DNA was quantitated using a Nanodrop 2000 spectrophotometer. DNA was kept frozen at -20 °C following extraction. A 710 base pair (bp) section of the CO1 gene was targeted for sequencing using the primers LCO1490/HCO2198 and polymerase chain reaction protocol put forth by Folmer et al. (1994). A 274 bp section of the H3 gene was targeted for sequencing using the H3F/H3R primers put forth by Colgan et al. (2000) and the following PCR protocol: 94 °C for 1 min, 40 cycles of 95 °C for 30 seconds, 55 °C for 30 seconds, and 72 °C for 30 seconds, followed by a final extension period of 72 °C for 7 min. Forward and reverse reads were obtained through GeneWiz (Azenta Life Sciences, South Plainfield, NJ). Each sequence was quality-assured, trimmed, and reverse reads were reverse-complemented using the BioEdit desktop software (v. 7.2.5; Hall 1999). Forward and reverse reads were then used to create one consensus sequence per individual.

For all phylogenetic analyses, sequences were input and aligned using ClustalW embedded within the MEGA-X environment (v. 10.0.1; Kumar et al. 2018). The Model Finder embedded within MEGA-X was used to find the best-fit substitution model based on the lowest Bayesian Information Criterion. All base positions with less than 95% site coverage were excluded from analyses. Bootstrap confidences of branch points were assessed using 10,000 bootstrap replicates within MEGA-X. Bayesian topologies and Bayesian posterior probabilities (BPP) of branch points were computed using the joint programs BEAUti (v. 1.10.4) and BEAST (v. 1.10.4) (Suchard et al. 2018). The maximum clade credibility tree was then selected from the BEAST output using TreeAnnotator (v. 1.10.4). The resulting figures were cleaned and finalized using FigTree (v. 1.4.4) (Rambaut 2018) and Adobe Illustrator (v. 27.3.1). All published gene sequences were downloaded from the National Center for Biotechnology Information (NCBI) nucleotide database. All alignments are freely available on Github (Repository: melissajbetters/CRM_Provanna).

To verify inclusion within the genus *Provanna*, we assessed our novel sequences in relation to other Abyssochrysoids including species in the genera *Abyssochrysos* (Tomlin, 1927), *Cordesia* (Warén & Bouchet, 2009), *Rubyspira* (Johnson et al., 2010), *Desbruyeresia* (Warén & Bouchet, 1993), *Alviniconcha* (Okutani & Ohta, 1988), and *Ifremeria* (Bouchet & Warén, 1991). The Vetigastropods *Caymanabyssiasolis* (Kano et al., 2016) and *Notocraterpustulosus* (Thiele, 1925) were used as the outgroup for investigations of CO1. The Vetigastropods *Lepetodriluspustulosus* (McLean, 1988) and *Pyropelta* sp. (McLean & Haszprunar, 1987) were used as outgroup for investigations of H3. To verify the specific identity of our specimens, we assessed our novel CO1 sequences in relation to all other *Provanna* species available on NCBI. While CO1 sequences exist for *P.annae* (Nekhaev, 2023), these were amplified using a primer set that targeted a different region of the CO1 gene from our novel sequences, thus precluding comparison. To account for intraspecific variation, a maximum of three sequences per species (chosen at random) were included in the tree. Additionally, gene sequences with tentative or unknown identities were also included in case our novel sequences matched these. *Desbruyeresiamelanioides* (Warén & Bouchet, 1993) was used as the outgroup.

To assess the robustness of current species delimitations within the genus, we calculated the average pairwise sequence divergence (APD) across CO1 sequences for *Provanna*. All sequences with verified species identities were included; Sequences with tentative or unknown species identities were excluded. Our novel sequences were assigned to their hypothesized species identities. All sequences were aligned using ClustalW embedded within MEGA-X and assessed using a Tamura 3-parameter substitution model (Tamura 1992), the pairwise deletion option (threshold = 95%), and 5,000 bootstrap replicates within MEGA-X. We then tested the number of species partitions supported within this dataset using the hierarchical clustering program ASAP (Assemble Species by Automatic Partitioning) (Puillandre et al. 2021).

### ﻿Key construction

**Figure 3:**
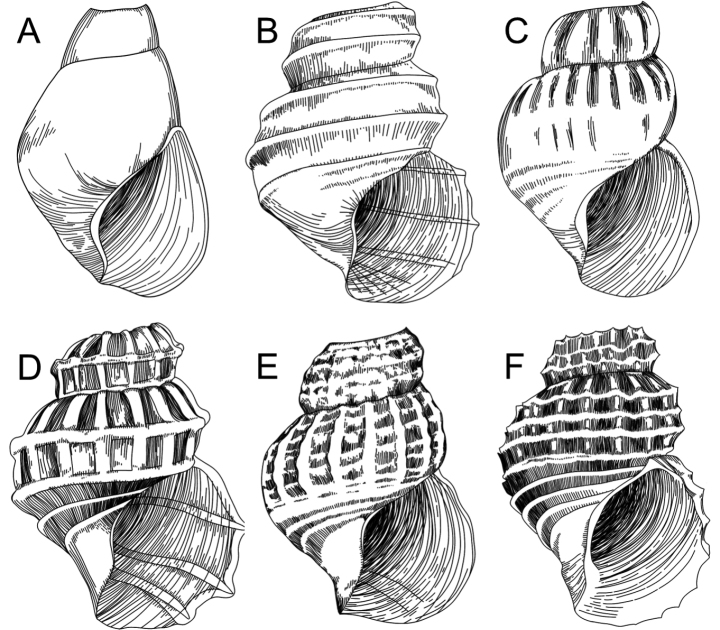
. Examples of *Provanna* shell morphological variety **A***P.kuroshimensis*, no sculpturing, growth lines present, flattened suture **B–F** constricted suture: **B***P.cooki*, spiral sculpture only, no sculptural elements **C***P.chevalieri*, axial sculpture only, no sculptural elements **D–F** both axial and spiral sculpturing: **D***P.fenestrata*, sculptures about equal in strength, no sculptural elements **E***P.clathrata*, axial sculpture stronger than spiral, blunt, sloping nodules **F***P.reticulata*, spiral sculpture stronger than axial, minor spines.

Using the conclusions drawn from the preceding sections, a taxonomic key for all genetically supported, extant species of *Provanna* was constructed. A polytomous key was chosen as the format to capture the natural variation found in *Provanna* shells (Fig. 3). Of the morphological characters annotated, we prioritized sorting shells based on aspects of shell sculpturing, as these characters are easily recognized and do not require additional processing to observe. Radular characteristics were only utilized within the key when no other shell character could discern between species. Several morphological characters were excluded from the key either because we determined that they would introduce too much subjectivity in responses (precluding consistent utility), overlapped among species, overlapped with other characters, or varied too much within species. Incorporating our own morphological results, the presence and number of basal ribs and penultimate whorl morphological characters were excluded. It is noted in the key where there is uncertainty in a species hypothesis which is then addressed in the Discussion.

## ﻿Results

### ﻿New occurrence records

**Superfamily Abyssochrysoidea**

**Family Provannidae**

**Genus *Provanna* (Dall, 1918)**

#### ﻿ Provanna laevis

Taxon classificationAnimaliaLittorinidaProvannidae

Warén & Ponder, 1991

26CE8C74-4B5D-5C30-93FF-C47BF8E5BBA9

##### New records.

Costa Rica • 25 specimens; Costa Rica Margin, Mound 12; 8.930°N, 84.313°W; 999 m; 22 May 2017; Lisa Levin, Charlotte Seid leg.; ALVIN Dive 4907, from wood; Scripps Benthic Invertebrate Collection (SBIC) M16112. 11 specimens; Costa Rica Margin, Mound 12; 8.930°N, 84.313°W; 1004 m; 25 May 2017; Greg Rouse, Todd Litke leg.; ALVIN Dive 4910, from rock; SBIC M16104 and M16106. 16 specimens; Costa Rica Margin, Mound 12; 8.929°N, 84.315°W; 964 m; 1 June 2017; Greg Rouse, Ben Moran leg.; ALVIN Dive 4917, from mussel shells; SBIC M16176. 89 specimens; Costa Rica Margin, Mound 12; 8.930°N, 84.313°W; 1003 m; 20 October 2018; Lisa Levin, Kyle Metcalfe leg.; ALVIN Dive 4974, from mussel shells; SBIC M16765. 78 specimens; Costa Rica Margin, Mound 12; 8.931°N, 84.313°W; 1004 m; 24 October 2018; Erik Cordes, Melissa Betters leg.; ALVIN Dive 4978, from mussel shells. 104 specimens; Costa Rica Margin, Mound 12; 8.931°N, 84.313°W; 1002–1004 m; 30 October 2018; Erik Cordes, Melissa Betters leg.; ALVIN Dive 4984, from mussel shells. 5 specimens; Costa Rica Margin, Mound 12; 8.930°N, 84.313°W; 1001 m; 31 October 2018; Erik Cordes, Melissa Betters leg.; ALVIN Dive 4985, from mussel shells. 475 specimens; Costa Rica Margin, Mound 12; 8.930°N, 84.312–84.313°W; 1002–1007 m; 2 November 2018; Erik Cordes, Melissa Betters leg.; ALVIN Dive 4987, from tubeworms. 6 specimens; Costa Rica Margin, Jaco Summit; 9.174°N, 84.800°W; 742 m; 6 January 2019; Greg Rouse, Allison Miller leg.; SUBASTIAN Dive 213, from wood; SBIC M17030. 793 specimens; Costa Rica Margin, The Thumb; 9.049°N, 84.354–84.394°W; 1071–1075 m; 10 January 2019; Erik Cordes, Melissa Betters leg.; SUBASTIAN Dive 217, from mussel shells. 22 specimens; Costa Rica Margin, The Thumb; 9.049°N, 84.354–84.394°W; 1071–1075 m; 10 January 2019; Erik Cordes, Melissa Betters leg.; SUBASTIAN Dive 217, from tubeworms.

##### Remarks.

The range of *P.laevis* is here expanded to three hydrocarbon seep locations at the CRM: Mound 12, Jaco Summit, and The Thumb. Their known distribution in the Eastern Pacific Ocean ranges from the Juan de Fuca Ridge to the Costa Rica Margin. Their known depth distribution in the Eastern Pacific Ocean is between 700–2000 m (Table 4).

**Table 4.** Summary of biogeographic information for each known species of *Provanna*. S = Seep, V = Vent, F = Organic Fall. Note that *P.glabra* is herein synonymized with *P.laevis* and *P.goniata* is herein synonymized with *P.ios*.

Species	Region(s)	Localities	Depth (m)	Habitat	Citations
*P.abyssalis* Okutani & Fujikura, 2002	W Pacific	Japan Trench	5379	S	Okutani and Fujikura 2002
*P.admetoides* Warén & Ponder, 1991	Gulf of Mexico	Off St. Petersburg, Gulf of Mexico	624–631	S	Warén and Ponder 1991
*P.annae* Nekhaev, 2023	N Pacific	Piip Volcano, Bering Sea	387–472		Nekhaev 2023; Rybakova et al. 2023
*P.beebi* Linse et al., 2019	Caribbean Sea	Beebe Vent Field, Mid-Cayman Spreading Center	4956–4972	V	Linse et al. 2019
*P.buccinoides* Warén & Bouchet, 1993	W Pacific	Hine Hina, Lau Basin; North Fiji Basin	1900–2765	V	Warén and Bouchet 1993
*P.chevalieri* Warén & Bouchet, 2009	E Atlantic	Regab, off West Africa	3150	S	Warén and Bouchet 2009
*P.cingulata* Chen et al., 2018	W Pacific	Shinkai Seep Field, Mariana Forearc	5687	S	Chen et al. 2018
*P.clathrata* Sasaki et al., 2016	W Pacific	Irabu Knoll, Hatoma Knoll, Yaeyama Knoll, Okinawa Trough; Haima seep, South China Sea; Manus Basin	1385–2190	V,S	Sasaki et al. 2016; Miyazaki et al. 2017; Poitrimol et al. 2022; He et al. 2023
*P.cooki* Linse et al., 2019	Southern	East Scotia Ridge, Southern Ocean	2396–2639	V	Linse et al. 2019
*P.exquisita* Chen & Watanabe, 2022	W Pacific	Eifuku Volcano, Mariana Arc	1606	V	Chen and Watanabe 2022
*P.fenestrata* Chen et al., 2019	W Pacific	Crane, Tarama Hill, Okinawa Trough; Sakai vent field; Haima seep, South China Sea	1385–1973	V,S	Chen et al. 2019; He et al. 2023
*P.ios* Warén & Bouchet, 1986 (Synonymous with: *P.goniata* Warén & Bouchet, 1986)	E Pacific	17 S, 13 N, 21 N, EPR; Guaymas Basin, Gulf of California; Galapagos Rift Zone; Costa Rica Margin	2000–2616	V,S	Warén and Bouchet 1986; Warén and Ponder 1991; Warén and Bouchet 2001; **This study**
*P.kuroshimensis* Sasaki et al., 2016	W Pacific	Kuroshima Knoll, off Okinawa	644	S	Sasaki et al. 2016
*P.laevis* Warén & Ponder, 1991 (Synonymous with: *P.glabra*Okutani et al., 1992)	W & E Pacific	Guaymas Basin, Gulf of California; Juan de Fuca; Oregon Margin; Off Hatsushima, Sagami Bay; Minami-Ensei Knoll, Iheya Ridge, Okinawa Trough; Costa Rica Margin	500–2004	V,S	Okutani et al. 1992; Warén and Ponder 1991; Okutani and Fujiwara 2000; Warén and Bouchet 2001; Fujikura et al. 2002; **This study**
*P.lomana* Warén & Bouchet, 1986	E Pacific	Off San Diego, off Point Dume, California; Oregon Margin; Off San Nicolas	450–1200	V,F	Warén and Bouchet 1986; Warén and Bouchet 2001; Smith and Baco 2003
*P.lucida* Sasaki et al., 2016	W Pacific	Minami-Ensei Knoll, Okinawa Trough	701	V	Sasaki et al. 2016
*P.macleani* Warén & Bouchet, 1989	E Pacific	Oregon Margin	2750	F	Warén and Ponder 2001
*P.muricata* Warén & Bouchet, 1986	E Pacific	21 N, East Pacific Rise; Galapagos Rift	2450–2615	V	Warén and Bouchet 1986; Warén and Ponder 1991
*P.nassariaeformis* Okutani, 1990	W Pacific	Snail Pit, Mariana Back-Arc Basin; Manus Basin	1912–3680	V	Okutani 1990; Wang et al. 2018
*P.pacifica* Warén & Bouchet, 1986	E Pacific	Gulf of Panama; Oregon Margin; Costa Rica Margin	1017–2750	F	Warén and Bouchet 1986; Warén and Bouchet 2001; **This study**
*P.reticulata* Warén & Bouchet, 2009	E Atlantic	Regab, off West Africa	3150	S	Warén and Bouchet 2009
*P.sculpta* Warén & Ponder, 1991	Gulf of Mexico	Off Louisiana, Gulf of Mexico	576	S	Warén and Ponder 1991; Warén and Bouchet 2001
*P.segonzaci* Warén & Ponder, 1991	W Pacific	Fiji Back-Arc; Hine Hina, Lau Basin	1750–1900	V	Warén and Ponder 1991; Warén and Bouchet 1993
*P.shinkaiae* Okutani & Fujikura, 2002	W Pacific	Japan Trench	5343	S	Okutani and Fujikura 2002
*P.stephanos* Chen et al., 2019	W Pacific	Off Hatsushima, Sagami Bay	860–908	S	Chen et al. 2019; Chen and Nomaki 2021
*P.subglabra* Sasaki et al., 2016	W Pacific	Hatoma Knoll, Izena Hole, Irabu Knoll, Minami-Ensei Knoll, Yaeyama Knoll, Okinawa Trough; Haima Seep, South China Sea	710–2190	V,S	Sasaki et al. 2016; Miyazaki et al. 2017; Xu et al. 2016; He et al. 2023
*P.variabilis* Warén & Bouchet, 1986	E Pacific	Endeavor Segment, Axial Seamount, Explorers Ridge, Juan de Fuca Ridge; Oregon Margin	1500–2927	V	Warén and Bouchet 1986; Warén and Bouchet 1993; Warén and Bouchet 2001

#### ﻿ Provanna ios

Taxon classificationAnimaliaLittorinidaProvannidae

Warén & Bouchet, 1986

0A9912AE-AE76-581D-BF5B-5BE3133A999F

##### New records.

Costa Rica • 42 specimens; Costa Rica Margin, Jaco Scar; 9.118°N, 84.839°W; 1757 m; 20 March 2017; Elena Perez, Geoff Cook leg.; ALVIN Dive 4874, from tubeworms; SBIC M12301. 3 specimens; Costa Rica Margin, Jaco Scar; 9.115°N, 84.836°W; 1834 m; 27 March 2017; Victoria Orphan, Kat Dawson leg.; ALVIN Dive 4912, from mussels; SBIC M16110 and M16127. 1 specimen; Costa Rica Margin, Jaco Scar; 9.116°N, 84.840°W; 1898 m; 28 March 2017; Greg Rouse, Jorge Cortes leg.; ALVIN Dive 4913, from tubeworms; SBIC M16144. 10 specimens; Costa Rica Margin, Jaco Scar; 9.117°N, 84.840°W; 1802 m; 29 March 2017; Chris Roman, Alanna Durkin leg.; ALVIN Dive 4914, from tubeworms; SBIC M16153, M16164, and M16166. 7 specimens; Costa Rica Margin, Jaco Scar; 9.117°N, 84.840°W; 1806 m; 17 October 2018; Erik Cordes, Rebecca Rutstein leg.; ALVIN Dive 4971, from rocks; SBIC M16730 and M16754. 1 specimen; Costa Rica Margin, Jaco Scar; 9.118°N, 84.840°W; 1803 m; 19 October 2018; Victoria Orphan, Natalya Gallo leg.; ALVIN Dive 4973, from mussels; SBIC M16724. 1 specimen; Costa Rica Margin, Jaco Scar; 9.118°N, 84.840°W; 1803 m; 19 October 2018; Victoria Orphan, Natalya Gallo leg.; ALVIN Dive 4973, from rock; SBIC M16741. 38 specimens; Costa Rica Margin, Jaco Scar; 9.118°N, 84.840°W; 1803 m; 23 October 2018; Erik Cordes, Joanna Klein leg.; ALVIN Dive 4977, from mussels; SBIC M16807. 37 specimens; Costa Rica Margin, Jaco Scar; 9.118°N, 84.841°W; 1780 m; 4 November 2018; Erik Cordes, Melissa Betters leg.; ALVIN Dive 4989, from tubeworms. 40 specimens; Costa Rica Margin, Jaco Scar; 9.117°N, 84.840°W; 1802–1812 m; 7 January 2019; Erik Cordes, Melissa Betters leg.; SUBASTIAN Dive 214, from mussels.

##### Remarks.

Detailed in full below, the specimens of *P.ios* presented here were previously referred to as *P.goniata* (Betters et al. 2023). The range of *P.ios* (inclusive of *P.goniata*), is here expanded to one seep locality at the CRM (Jaco Scar). Their range now includes the sites 17S, 13N, and 21N at the East Pacific Rise, the Guaymas Basin, the Galapagos Rift Zone, and the Costa Rica Margin between 2000–2616 m depth (Table 4).

#### ﻿ Provanna pacifica

Taxon classificationAnimaliaLittorinidaProvannidae

Warén & Bouchet, 1986

37B342CF-0F2B-592E-A8AA-E9E811A983D1

##### New records.

Costa Rica • 3 specimens; Costa Rica Margin, Quepos Seep; 9.031°N, 84.619°W; 1413 m; 7 June 2017; Lisa Levin, Kris Krasnosky leg.; ALVIN Dive 4924, from mussels; SBIC M16204. 6 specimens; Costa Rica Margin, Mound 11; 8.922°N, 84.305°W; 1017 m; 3 November 2018; Victoria Orphan, Hang Yu leg.; ALVIN Dive 4988, on wood; SBIC 16955.

##### Remarks.

The range of *P.pacifica* is here expanded to one hydrocarbon seep (Quepos Seep) and one organic fall at the CRM (Mound 11). Their occurrence here on Bathymodiolin mussels represents the first time they have been observed as, potentially, permanent denizens of a hydrocarbon seep environment. Their distribution now includes the Oregon Margin, the Costa Rica Margin, and the Gulf of Panama between 1017–2750 m depth (Table 4).

### ﻿Morphological analysis

**Figure 4.**
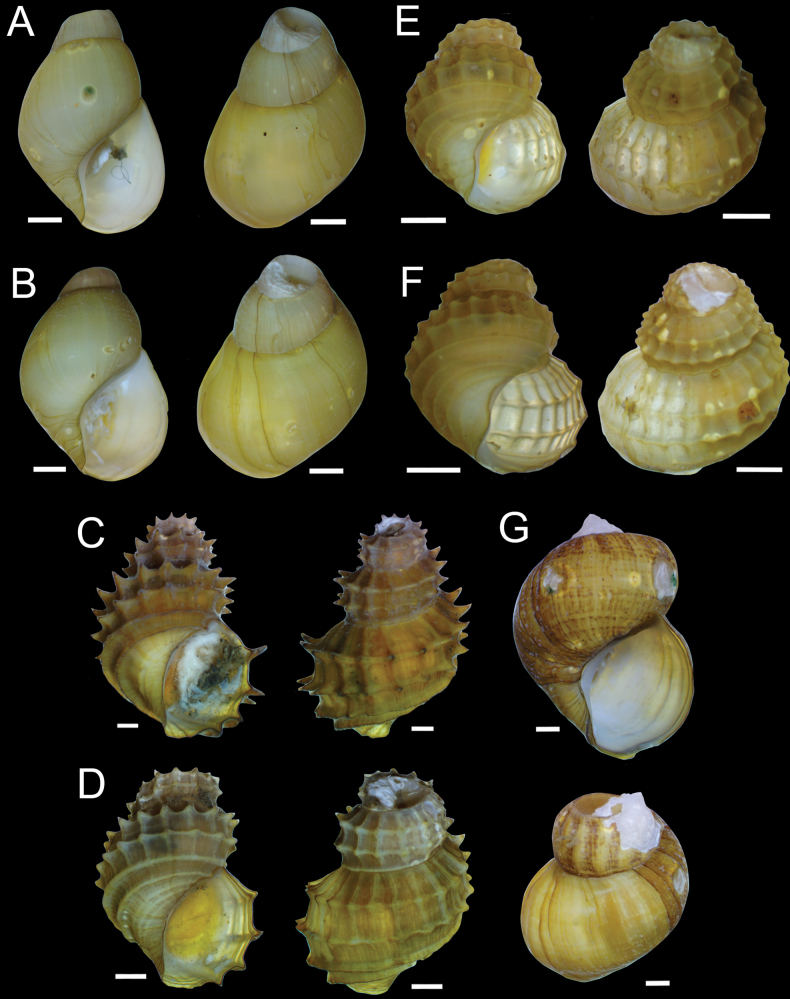
*Provanna* morphotypes sampled from the CRM**A, B***P.laevis* from mussel shells, Mound 12, AD4917, 965 m **C, D***P.ios* from unknown substrate, Jaco Scar, SD214, 1803 m **E** Specimens of *P.pacifica* from sunken wood, Mound 11, AD4988, 1017 m **F** Specimen of P.cf.lomana from mussel shells, Quepos Seep, AD4924, 1413 m. Both the dorsal and ventral view of each shell is shown. Scale bars: 1 mm.

**Figure 5.**
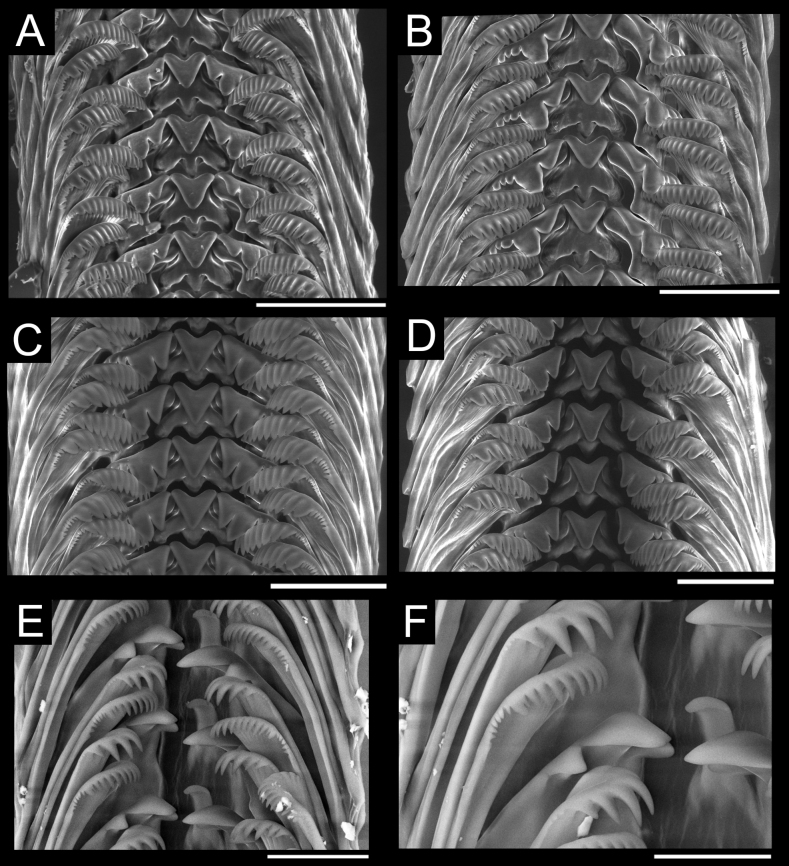
Representative radulae of Costa Rican *Provanna* species **A***P.laevis* from mussel shells **B***P.laevis* from unknown substrate **C***P.ios* from mussel shells **D***P.ios* from unknown substrate **E, F***P.pacifica* from wood. Scale bars: 20 µm (**F**); 30 µm (**E**); 40 µm (**C**); 50 µm (**A, B, D**).

In total, 1,817 *Provanna* specimens were sampled from six sites at the CRM (see Table 1 for details). All specimens were sorted into one of four distinct morphotypes and subsequently assigned the following species identities: *Provannalaevis* (*n* = 1624) sampled from Jaco Summit, The Thumb, and Mound 12, *Provannaios* (*n* = 180) sampled from Jaco Scar, *Provannapacifica* (*n* = 9) sampled from Quepos Seep and Mound 11, and Provannacf.lomana (*n* = 2) sampled from Mound 11 (Fig. 4). *Provannalaevis* was identified as it is the only smooth-shelled species from the Eastern Pacific. Our specimens of *P.ios* fit most closely the morphological description of *P.goniata* and were originally designated as such (Warén and Bouchet 1986; Betters et al. 2023) However, given our genetic results (detailed in the Results section Genetic Analysis), we amend this original identification to *P.ios* and address the validity of *P.goniata* as a distinct species in the Discussion. Specimens of *P.pacifica* were identified based on their sculpturing, their relatively small size, and the fact that this species was originally described from a soft-bottom, low-productivity seep in the Gulf of Panama very similar to its habitat at Costa Rica (Warén and Bouchet 1986). Provannacf.lomana was tentatively identified by its unique feature of having only axial sculpturing on its body whorl. Of these specimens, a total of 158 representative *Provanna* covering the full geographic, temporal, and size range of each morphotype were measured (*P.laevis*, *n* = 96; *P.ios*, *n* = 52; *P.pacifica*, *n* = 8; and P.cf.lomana, *n* = 2). Representative radulae were successfully extracted and imaged for all species except P.cf.lomana (Fig. 5).

**Figure 6.**
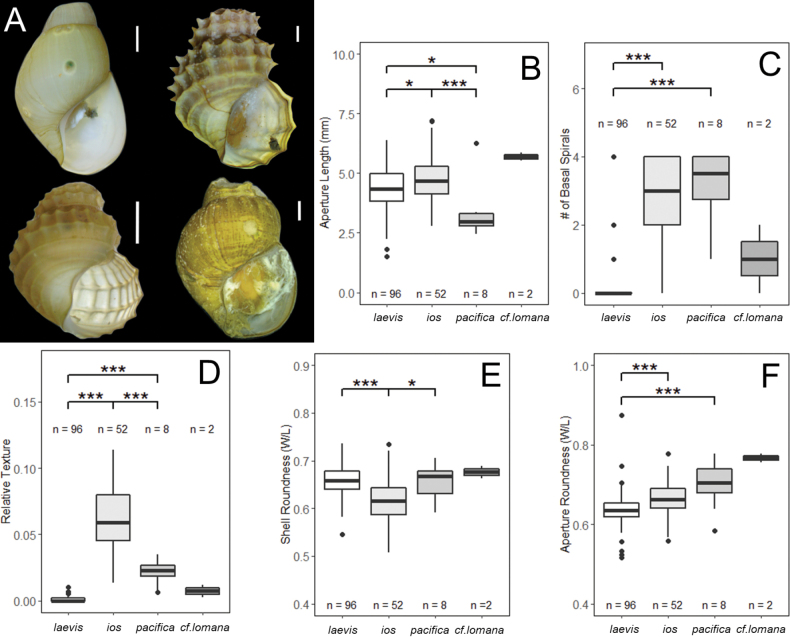
A the four morphotypes sampled **B–F** comparison of morphological traits among each morphotype. The number of individuals represented on the graph and included in one-way ANOVAs are denoted by “*n* =” above or below each bar. Resultant p-values from one-way ANOVAs are denoted above each graph (p-value: 0 < *** < 0.001 < ** < 0.01 < * 0.05). Provannacf.lomana was excluded from all ANOVAs due to the small number of individuals, but is included here for graphical comparison. Note that the graphs of Shell Roundness **E** and Aperture Roundness **F** have y-axes that do not start at zero. Scale bars: 1 mm.

Across all Costa Rican specimens, measurements of shell length, shell width, aperture length, and aperture width showed significant collinearity (PCC > +0.95, all pairs). Because the whorls past the body whorl showed variable levels of degradation, shell size was represented in analyses by aperture length alone, as we had more confidence in this measurement. All species sampled were comparable in size, with *Provannaios* being the largest and *P.pacifica* being the smallest (Fig. 6B). The number of basal ribs, despite being commonly used to describe *Provanna* species, showed variation across all morphotypes (Fig. 6C). The difference in relative texture between *P.ios* and *P.pacifica* was significant (p < 0.001), supporting the utility of their sculptural elements in delineating species (Fig. 6D). In general, shells with granules longer than 3% of the shell length (major spines) were reliably *P.ios*. *Provannaios* had the most oblique shell shape overall (Fig. 6E) and *P.laevis* had the most oblique aperture shape overall (Fig. 6F).

### ﻿Genetic analysis

CO1 sequences were obtained from our specimens of *P.laevis* (*n* = 4), *P.ios* (*n* = 2), and *P.pacifica* (*n* = 2). All efforts to amplify CO1 for specimens of P.cf.lomana were unsuccessful. CO1 sequences generated were uploaded to GenBank and assigned accession numbers (OM914402–OM914408 & OP577954). H3 sequences were obtained from our specimens of *P.laevis* (*n* = 1), *P.ios* (*n* = 2), *P.pacifica* (*n* = 2), and P.cf.lomana (*n* = 1). H3 sequences generated were uploaded to GenBank and assigned accession numbers (OR687645–OR687650).

**Figure 7.**
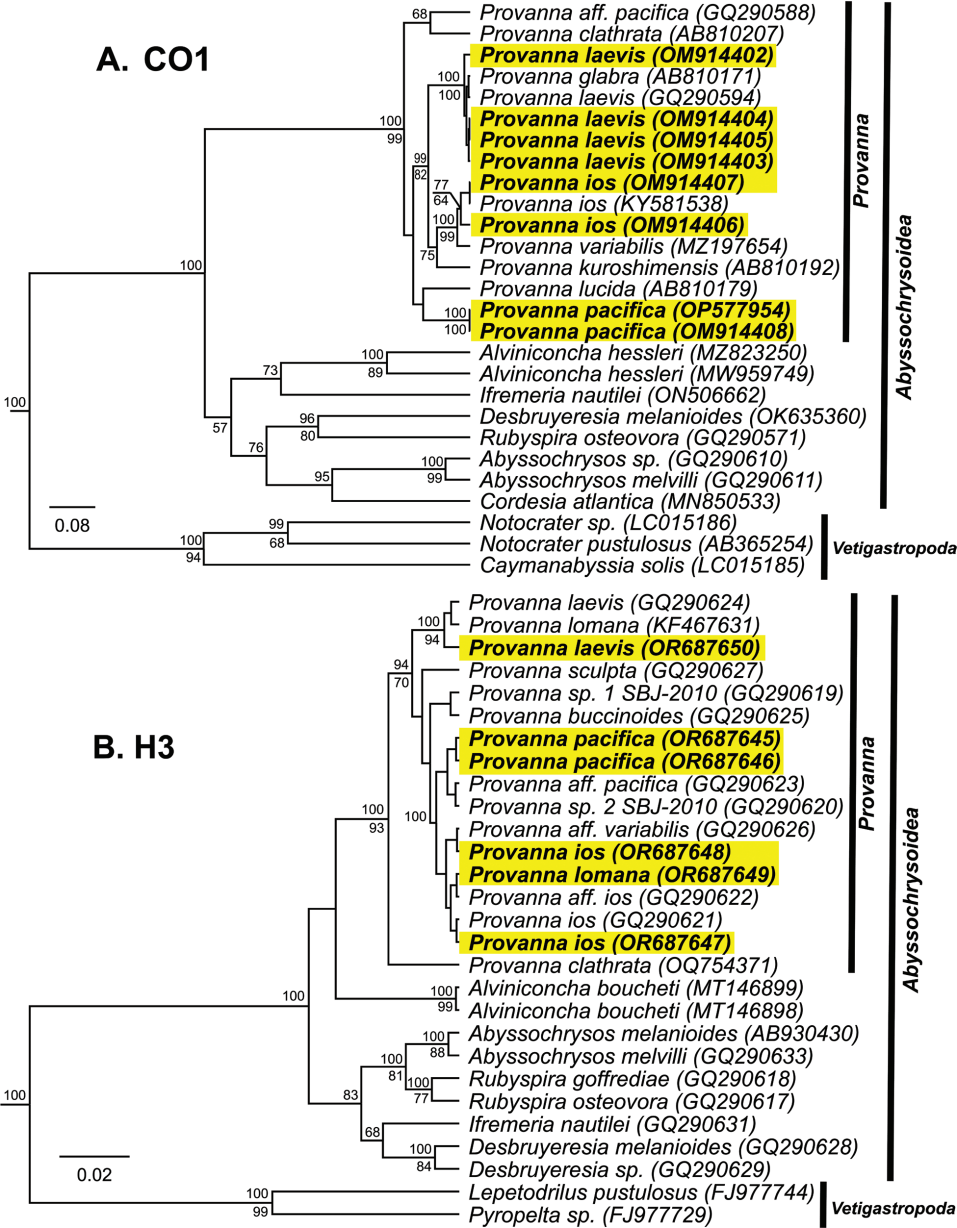
Bayesian topology of Abyssochrysoid gastropod mollusks **A** topology based on a 449 bp region of the mitochondrial CO1 gene. Topology was inferred using the HKY+G+I substitution model **B** topology based on a 266 bp region of the nuclear H3 gene. Novel sequences are bolded and highlighted in yellow. Numbers above branch nodes represent Bayesian posterior probabilities. Numbers below branch nodes represent the proportion of replicate trees in which the associated taxa clustered together in the bootstrap test (10,000 replicates). Only values above 50% are shown. The tree is drawn to scale, with branch lengths representing the number of base substitutions accumulated over time.

**Figure 8.**
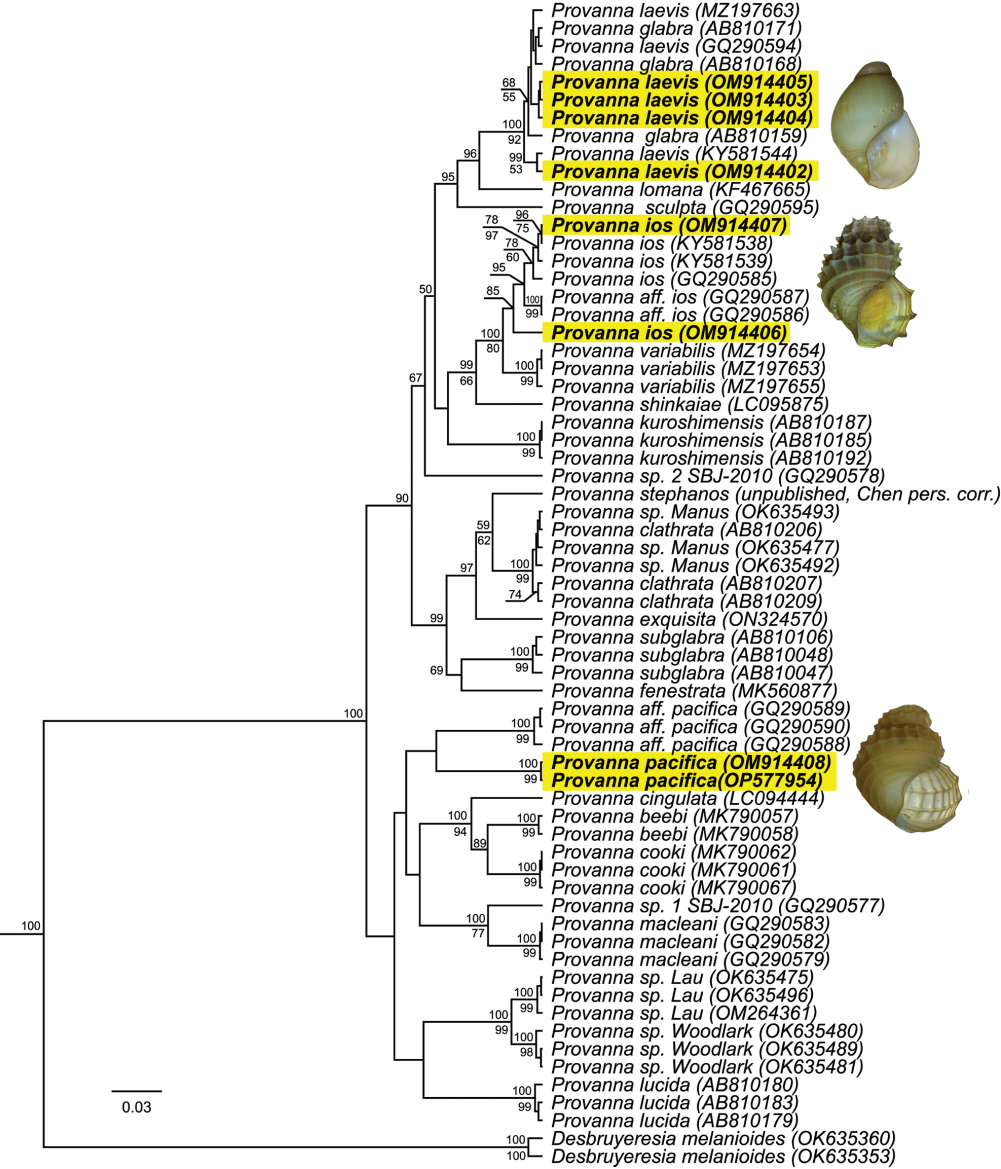
Bayesian phylogenetic tree of *Provanna* based on a 452 bp region of the mitochondrial CO1 gene. Novel sequences are bolded and highlighted in yellow. Topology was inferred using the HKY+G+I substitution model. Numbers above branch nodes represent Bayesian posterior probabilities. Numbers below branch nodes represent the proportion of replicate trees in which the associated taxa clustered together in the bootstrap test (10,000 replicates). Only values above 50 are shown. The tree is drawn to scale, with branch lengths representing the number of base substitutions accumulated over time.

Phylogenetic analyses support the inclusion of our specimens in the genus *Provanna* with high confidence for CO1 (Bayesian Posterior Probability (BPP) = 100, ML = 90) (Fig. 7A) and H3 (BPP = 100, ML = 93) (Fig. 7B). Species-level investigations showed that sequences from the same *Provanna* species nested together and away from others on the tree (Fig. 8). Our specimens of *P.laevis* nested among *P.laevis* and *P.glabra* with high confidence and little to no distinction (BPP = 100, Bootstrap = 92). Despite our samples matching the physical description of *P.goniata*, these grouped together with sequences of *P.ios* and P.aff.ios with moderate confidence (BPP = 85). These were, however, all delineated as the sister group to *P.variabilis* (BPP = 100, Bootstrap = 80). Our specimens of *P.pacifica* grouped together and away from all other species on the tree (BPP = 100, Bootstrap = 99), though they were most closely related to P.aff.pacifica. However, given that *P.pacifica* has never been barcoded before, and that our specimens closely match the physical description and geographic distribution of the species proper, we assert that our specimens are indeed *P.pacifica*.

Sequences of unknown identity (*Provanna* sp. 1 (GQ290577) and *Provanna* sp. 2 (GQ290578)) did not group together with any known species on the tree. Sequences from the Manus Basin that were previously identified as *P.clathrata* (Poitrimol et al. 2022) nested with high confidence among sequences of *P.clathrata* from the Okinawa Trough (Sasaki et al. 2016) (BPP = 100, Bootstrap = 99), confirming this identification and the range expansion for this species (see Table 4). As previously found, sequences from the Woodlark and Lau Basins group apart from all other known species on tree, as well as each other (Poitrimol et al. 2022) (BPP = 100, Bootstrap = 99). However, more detailed morphological investigations are needed to address whether these represent *P.buccinoides*, *P.segonzaci*, or one or more new species.

Average pairwise sequence divergences (APD) were computed across CO1 sequences (*n* = 236) (Table 5). APD calculations confirmed *Provanna* species as being more closely related to each other than to the outgroup (ingroup < 0.2 < outgroup). Almost all APD calculations fell between 0.05–0.13, confirming robust species distinctions overall within this genus (Hebert et al. 2003; Johnson et al. 2008). One exception to this was *P.glabra* and *P.laevis*, which showed very low sequence divergence (APD = 0.01, SE = 0.00). *Provannavariabilis* also appeared closely related to *P.ios* (APD = 0.04, SE = 0.01), supporting the topology from Fig. 8 designating them as sister clades. Comparisons within species were limited to those with more than one representative CO1 sequence, thus excluding *P.cingulata*, *P.cooki*, *P.exquisita*, *P.fenestrata*, *P.lomana*, *P.sculpta*, *P.shinkaiae*, and *P.stephanos*. Of the remaining species, average within-species APD’s fell below 0.03, confirming that intraspecific divergence was lower than interspecific divergence across *Provanna* species.

**Table 5.** Genetic distance estimates among and within known *Provanna* species. Distances were calculated using the Tamura 3-parameter model and 5,000 bootstrap replicates. Numbers below central margin represent the number of base substitutions per site, averaging over all sequence pairs. Numbers above central margin represents standard errors. The central margin represents within-group genetic distances. Calculations were conducted using 236 total nucleotide sequences. Outgroup = *Desbruyeresiamelanioides*.

*Provanna*	1	2	3	4	5	6	7	8	9	10	11	12	13	14	15	16	17	18	19	20
*1. beebi*	0.00	*0.01*	*0.02*	*0.01*	*0.02*	*0.02*	*0.02*	*0.02*	*0.02*	*0.02*	*0.02*	*0.02*	*0.02*	*0.01*	*0.02*	*0.02*	*0.01*	*0.02*	*0.02*	*0.03*
*2. cingulata*	0.06	NA	*0.02*	*0.01*	*0.03*	*0.02*	*0.02*	*0.02*	*0.02*	*0.02*	*0.02*	*0.02*	*0.02*	*0.02*	*0.02*	*0.02*	*0.02*	*0.02*	*0.02*	*0.03*
*3. clathrata*	0.11	0.12	0.01	*0.02*	*0.02*	*0.01*	*0.02*	*0.02*	*0.02*	*0.02*	*0.02*	*0.02*	*0.02*	*0.02*	*0.02*	*0.02*	*0.01*	*0.01*	*0.02*	*0.03*
*4. cooki*	0.06	0.06	0.11	NA	*0.02*	*0.02*	*0.02*	*0.02*	*0.02*	*0.02*	*0.02*	*0.02*	*0.02*	*0.02*	*0.02*	*0.02*	*0.02*	*0.02*	*0.02*	*0.03*
*5. exquisita*	0.09	0.11	0.08	0.09	NA	*0.02*	*0.02*	*0.02*	*0.02*	*0.02*	*0.02*	*0.03*	*0.03*	*0.02*	*0.02*	*0.03*	*0.02*	*0.02*	*0.02*	*0.05*
*6. fenestrata*	0.11	0.11	0.08	0.10	0.08	NA	*0.02*	*0.02*	*0.01*	*0.02*	*0.01*	*0.02*	*0.02*	*0.02*	*0.02*	*0.02*	*0.01*	*0.02*	*0.02*	*0.03*
*7. glabra*	0.09	0.12	0.11	0.10	0.08	0.09	0.01	*0.01*	*0.02*	*0.00*	*0.01*	*0.02*	*0.02*	*0.02*	*0.02*	*0.02*	*0.02*	*0.02*	*0.01*	*0.04*
*8. ios*	0.10	0.11	0.10	0.11	0.08	0.10	0.08	0.02	*0.01*	*0.01*	*0.01*	*0.02*	*0.02*	*0.02*	*0.01*	*0.01*	*0.02*	*0.02*	*0.01*	*0.04*
*9. kuroshimensis*	0.10	0.11	0.11	0.09	0.10	0.08	0.08	0.08	0.01	*0.02*	*0.01*	*0.02*	*0.02*	*0.01*	*0.02*	*0.02*	*0.02*	*0.02*	*0.01*	*0.03*
*10. laevis*	0.09	0.11	0.10	0.10	0.08	0.09	0.01	0.07	0.08	0.01	*0.01*	*0.02*	*0.02*	*0.02*	*0.01*	*0.02*	*0.02*	*0.02*	*0.01*	*0.04*
*11. lomana*	0.10	0.10	0.10	0.08	0.07	0.08	0.06	0.08	0.07	0.07	NA	*0.02*	*0.02*	*0.02*	*0.01*	*0.02*	*0.02*	*0.02*	*0.01*	*0.03*
*12. lucida*	0.13	0.12	0.11	0.11	0.12	0.12	0.14	0.13	0.11	0.14	0.12	0.00	*0.02*	*0.02*	*0.02*	*0.02*	*0.02*	*0.02*	*0.02*	*0.03*
*13. macleani*	0.09	0.10	0.11	0.09	0.10	0.10	0.09	0.11	0.09	0.09	0.11	0.12	0.00	*0.02*	*0.02*	*0.02*	*0.02*	*0.02*	*0.02*	*0.03*
*14. pacifica*	0.08	0.09	0.08	0.09	0.08	0.08	0.09	0.09	0.08	0.09	0.08	0.10	0.09	0.00	*0.01*	*0.02*	*0.01*	*0.02*	*0.02*	*0.03*
*15. sculpta*	0.09	0.10	0.08	0.09	0.06	0.08	0.08	0.08	0.08	0.08	0.06	0.13	0.11	0.08	NA	*0.02*	*0.01*	*0.02*	*0.01*	*0.03*
*16. shinkaiae*	0.09	0.11	0.10	0.11	0.10	0.11	0.09	0.06	0.09	0.09	0.09	0.11	0.11	0.10	0.09	NA	*0.02*	*0.02*	*0.01*	*0.04*
*17. stephanos*	0.09	0.10	0.04	0.10	0.05	0.07	0.10	0.08	0.09	0.09	0.08	0.10	0.10	0.07	0.07	0.10	NA	*0.01*	*0.01*	*0.03*
*18. subglabra*	0.10	0.10	0.07	0.11	0.08	0.09	0.10	0.10	0.12	0.10	0.09	0.14	0.11	0.12	0.09	0.11	0.08	0.01	*0.02*	*0.03*
*19. variabilis*	0.11	0.11	0.11	0.11	0.08	0.10	0.08	0.04	0.08	0.08	0.08	0.13	0.11	0.10	0.08	0.07	0.09	0.11	0.03	*0.03*
*20. Outgroup*	0.23	0.24	0.22	0.25	0.24	0.24	0.26	0.27	0.24	0.26	0.25	0.24	0.22	0.25	0.25	0.28	0.24	0.24	0.26	0.01

Hierarchical clustering performed by ASAP yielded 14 discreet subsets from an input of 19 hypothesized species (*n* = 236 sequences, p < 0.0001): (1) *P.glabra*-*P.laevis*, (2) *P.variabilis*-*P.ios*, (3) *P.pacifica*, (4) *P.fenestrata*, (5) *P.cooki*, (6), *P.beebi*, (7), *P.cingulata*, (8) *P.exquisita*-*P.stephanos*-*P.clathrata*, (9) *P.sculpta*, (10) *P.lomana*, (11) *P.macleani*, (12) *P.subglabra*, (13) *P.kuroshimensis*, (14) *P.lucida*. The threshold distance (Dt) used to partition the samples into species was 0.0496 (p < 0.0001) and the most common genetic distance between sequence pairs fell between 0.09–0.1.

### ﻿Polytomous key for species identification

**Table:** 

1	Only axial sculpturing	**2**
–	Only spiral sculpturing	**3**
–	Both axial and spiral sculpturing	**4**
–	No sculpturing present	**5**
2	Axial sculpture extends to the posterior end of the aperture, but not into the basal area	***P.lomana***
–	Axial sculpture does not extend to the posterior end of the aperture, instead stopping midway down the body whorl	***P.chevalieri***
3	Shell is thin and translucent; One can easily see through the shell	**6**
–	Shell is not noticeably translucent; One cannot easily see through the shell	**7**
4	Sculptural elements absent	**9**
–	Sculptural elements present	**10**
5	Sculptural elements present	***P.beebi***
–	Sculptural elements absent	**26**
6	1–3 spiral ribs on the body whorl above the posterior end of the aperture	***P.lucida***
–	4 or more spiral ribs on the body whorl above the posterior end of the aperture	***P.cingulata***
7	Central radular teeth highly diminished, being very narrow with a very truncated, cusp (see Fig. 2C for definitions)	***P.macleani***
–	Central radular teeth broad with a truncated cusp and a flat or rounded anterior ridge	***P.beebi***
–	Central radular teeth broad with a very short cusp and concave anterior ridge	***P.reticulata***
–	Central teeth are typical of genus with a triangular cusp	**8**
8	First lateral teeth have long, lobate major denticles and an obtuse buttress angle (see Fig. 2C for definitions)	***P.cooki* (see Discussion)**
–	First lateral teeth have long, triangular major denticles and an acute buttress angle	***P.variabilis* (see Discussion)**
9	Axial and spiral sculptures are strong, raised, and equally spaced, creating a regular lattice-like sculpture (see Fig. 3D for example	**11**
–	Regular, lattice-like sculpture is not formed	**12**
10	Sculptural elements are major spines (see Fig. 2A for definitions)	**13**
–	Sculptural elements are minor spines	**14**
–	Sculptural elements are punctuated, rounded beads	**15**
–	Sculptural elements are blunt and sloping nodules	**16**
11	There are more than 30 axial ribs on the body whorl and 2–3 spiral ribs	***P.admetoides***
–	There are 15–20 spiral ribs on the body whorl and 1–2 spiral ribs	***P.fenestrata***
12	Central radular teeth highly diminished and narrow with a very truncated, cusp (see Fig. 2C for definitions)	***P.macleani***
–	Central radular teeth broad with a short cusp and a flattened anterior ridge	***P.chevalieri***
13	Major spines on the second or third spiral rib on the body whorl connect at times to form a flattened shelf or keel	**17**
–	Major spines do not obviously connect nor do they form a flattened keel	**18**
14	There are more than 30 axial ribs on the body whorl, forming a regular, lattice-like sculpture	***P.admetoides***
–	There are fewer than 30 axial ribs on the body whorl	**19**
15	Shell globose; Shell roundness (Shell Width / Truncated Length) (see Fig. 2B) > 0.65	***P.nassariaeformis***
–	Shell not globose; Shell roundness < 0.65	**22**
16	Axial and spiral sculptures are strong, evenly raised, and equally spaced, creating a regular lattice-like sculpture (see Fig. 3D for example)	***P.fenestrata***
–	Axial and spiral sculptures vary in strength across the body whorl; Regular, lattice-like sculpture is not formed	**23**
17	Axial ribs form clear cords that are present along the entire body whorl, rectangular lattice-like sculpturing formed (see Fig. 3D for example)	***P.exquisita***
–	Axial ribs vary in strength along the body whorl, sometimes disappearing entirely, no clear lattice-like sculpturing formed	***P.stephanos***
18	Shell is very slender; Shell roundness (Shell Width / Truncated Length) (see Fig. 2B) < 0.55	***P.shinkaiae* (see Discussion)**
–	Shell roundness > 0.55	***P.ios* (previously *P.goniata*) (see Discussion)**
19	Central radular teeth highly diminished and narrow with a very truncated, cusp (see Fig. 2C for definitions)	***P.pacifica***
–	Central radular teeth broad with a very short, blunt cusp	***P.reticulata***
–	Central radular teeth are typical of genus with a triangular cusp	**20**
20	Anterior end of aperture has a round, globose shape	***P.muricata***
–	Anterior end of aperture has elongated, tapering shape	**21**
21	First lateral teeth have major denticles that are notched on the internal edge and rounded (see Fig. 2C for definitions)	***P.segonzaci***
–	First lateral teeth have major denticles that are not notched	**22**
22	Shell is globose; Shell roundness (Shell Width / Truncated Length) (see Fig. 2B) ≥ 0.6	***P.clathrata* (see Discussion)**
–	Shell is slender; Shell roundness ≤ 0.55	***P.ios* (previously *goniata*) (see Discussion)**
23	Sculptural elements appear as small pockmarks and extend to the anterior end of the shell	***P.beebi***
–	Sculptural elements appear as flat lines of beads arranged longitudinally, stopping abruptly at the first basal rib	***P.sculpta***
24	Axial sculpture varies in strength across the body whorl, sometimes disappearing entirely or extending only part of the way down the body whorl	***P.variabilis***
–	Axial sculpture is strong and even along the body whorl	**25**
25	Central radular teeth are highly diminished and narrow with a very truncated, cusp (see Fig. 2C for definitions)	***P.pacifica***
–	Central radular teeth are broad with blunt cusps and rounded or flat anterior ridges	***P.buccinoides***
–	Central radular teeth are typical of genus with triangular cusps	**26**
26	First lateral teeth have major denticles that are notched interiorly and lobate (see Fig. 2C for definitions)	***P.segonzaci***
–	Marginal teeth alternate in form between having 15–20 denticles	***P.muricata***
–	Marginal teeth all have between 9–10 denticles	***P.clathrata***
27	Shell is thin and translucent, one can easily see the body through the shell	**28**
–	Shell is not noticeably translucent	**29**
28	Central radular teeth have a long, triangular cusp; first lateral teeth have major denticles that are long and lobate (see Fig. 2C for definitions)	***P.annae* (see Discussion)**
–	Central radular teeth have a short, triangular cusp; first lateral teeth have major denticles that are long and triangular	***P.lucida* (see Discussion)**
29	Shell suture is highly constricted, giving the whorls an inflated, rounded appearance	**30**
–	Shell suture is not highly constricted	**31**
30	Shell roundness (Shell Width / Truncated Length) (see Fig. 2B) ~ 0.5	***P.abyssalis***
–	Shell roundness ~ 0.6	***P.cooki***
31	First lateral teeth have very truncated, short denticles	***P.laevis***
–	First lateral teeth have long major denticles	**32**
32	Central teeth have short, blunt cusps; lateral teeth have major denticles that are long, lobate (see Fig. 2C for definitions)	***P.laevis* (previously *P.glabra*)**
–	Central teeth have long, sharp, triangular cusps; lateral teeth have major denticles that are long, lobate	***P.kuroshimensis* (see Discussion)**
–	Central teeth have long, sharp, triangular cusps; lateral teeth have major denticles that are long, sharp	***P.subglabra* (see Discussion)**

## ﻿Discussion

This study presents new records and gene sequences for *P.laevis*, *P.ios*, *P.pacifica*, and P.cf.lomana from the Costa Rica Margin. Integrating these novel morphological and genetic data, we review the distinction among species and present the first polytomous identification key for the genus *Provanna*. In both our morphological and genetic investigations, similarities among species were revealed and are discussed below.

### ﻿Cryptic species

Several *Provanna* species show little to no morphological distinction. For example, certain shell morphotypes of *P.clathrata* and *P.ios* have no discernable differences from one another besides the number of denticles on their outer marginal teeth (*P.clathrata* have about ten while *P.ios* have about 20) (Table 3). Certain shell morphotypes of *P.ios* and *P.shinkaiae* may also resemble one another. Yet, the lobate major denticles of *P.shinkaiae*’s first lateral teeth distinguish it from *P.ios*. Depending on morphotype, *P.cooki* and *P.variabilis* may also display criticism. Both may have no axial ribs, three spiral ribs on the body whorl, and no sculptural elements. However, they may be distinguished by the shape of the major denticles of their first lateral teeth (Table 3). Finally, *P.annae* and *P.lucida* both have unsculptured, translucent shells with constricted sutures. Their distinguishing feature is the shape of the major denticles of their first lateral teeth (*P.annae* are lobate, *P.lucida* are triangular). Regardless of the reliability of these radular characteristics, each of these species pairs may also be readily distinguished through CO1 barcoding (Table 5) (Nekhaev 2023).

The smooth-shelled species *P.laevis*, *P.glabra*, *P.kuroshimensis*, and *P.subglabra* are also morphologically indistinguishable based on shell characters (Table 3). All species have no sculpturing, slender shells, and a flattened suture. *Provannasubglabra* may be distinguished from the other three by having long, sharp major denticles on its first lateral teeth. Similarly, *P.laevis* should be distinguishable by having truncated, lobate denticles on its first lateral teeth and its location in the Eastern Pacific. Contrary to expectations, the radulae of our specimens from the CRM closely resembled that of *P.glabra* or *P.kuroshimensis* with long, lobate major denticles (Fig. 5A, B). Genetic characterization could not distinguish *P.laevis* from *P.glabra*, but could readily distinguish *P.kuroshimensis* (Fig. 8, Table 5).

Specimens collected from the Costa Rica Margin revealed that not all shell characters are useful in delineating species. Despite its widespread use in taxonomic descriptions, the number of basal ribs showed notable variation within species. Furthermore, as basal ribs are often very weak and difficult to count consistently, these were not used as a taxonomically informative characters in the key, nor do we recommend their use in distinguishing species in the future.

### ﻿Species delimitation

Our genetic investigations supported most current taxonomic delimitations, finding robust genetic distances among the 19 species from which CO1 sequences exist. Nonetheless, automatic partitioning based on CO1 supported the consolidation of several species. *Provannavariabilis* and *P.ios*, for example, were not partitioned. However, as these species are distinguished in both our phylogenetic analyses as well as by their morphological characteristics, more data are needed to verify this genetic similarity before taxonomic revision is undertaken. Similarly, the species *P.exquisita*, *P.stephanos*, and *P.clathrata* were also not partitioned. However, as these are also distinguished in our phylogenetic analyses and by their shell and radular characteristics, we believe more data are needed to warrant collapse.

*Provannalaevis* from the Eastern Pacific and *P.glabra* from the Western Pacific exhibited significant genetic overlap in our species-level phylogeny (Fig. 8), our distance matrix (Table 5) and were not distinguished during automatic partitioning. This similarity has been noted by previous studies (Sasaki et al. 2016; Linse et al. 2019). Given that CO1 seems informative for the rest of this genus, this similarity is noteworthy. Both *P.laevis* and *P.glabra* inhabit similar depth ranges, a variety of chemosynthesis-based environments, and are morphologically indistinct in all but radular morphology (Warén and Bouchet 1986; Okutani et al. 1992), which may not be sufficient to indicate complete lineage sorting. They have typically been distinguished by their distributions. Nevertheless, there may be numerous, undiscovered chemosynthetic sites, including large biomass falls (e.g., whale falls) that could provide the stepping stones necessary for connectivity across the Pacific. Furthermore, Pacific northern equatorial and subsurface counter currents may transport upper and lower layers of water, respectively, from west to east along the 7°N latitude (Kessler 2006), coinciding neatly with the region of study in Costa Rica (~ 8–9°N). Conversely, water upwelled at the Costa Rica Dome may flow east across the Pacific via the Northern Equatorial Current (Kessler 2006) and into the North Pacific Gyre, where it may realistically encounter the Eastern shores of Japan and even the Western shores of North America. Recent work supports the highly adaptable nature of *P.laevis*, which may explain a very broad distribution (Betters et al. 2023). Given these morphological, genetic, and biogeographic data, collected over several independent studies (Sasaki et al. 2016; Linse et al. 2019; Betters et al. 2023) and supported once again here, it is thus recommended that *P.glabra* and *P.laevis* be considered one species. As *P.glabra* is the younger name, we here synonymize it with *P.laevis*, as per the International Code of Zoological Nomenclature Article 23.1. These species are thus treated as synonymous hereafter.

Specimens of *P.ios* from the CRM were originally identified as *P.goniata*, given that their shells are decorated with major spines rather than minor spines (Fig. 4C, D) and that they are found at hydrocarbon seeps, rather than that at hydrothermal vents (Betters et al. 2023). We first reconsidered this identification when genetic barcoding of both CO1 and H3 could not reliably distinguish these specimens from *P.ios* (Figs 7, 8, Table 5). Furthermore, recent work has found that when specimens at the CRM were sampled from higher concentrations of hydrocarbons and sulfides, they tended to have more slender, thinner, and larger shells (Betters et al. 2023). This means that as they inhabit more vent-like conditions, they resemble more closely the vent species *P.ios* (Table 3). Thus, it is highly likely that these two species are actually ecotypes of a single molecular taxonomic unit, where *P.ios* is the vent ecotype and *P.goniata* is the seep ecotype. Additionally, both species are found at similar depths in the Eastern Tropical Pacific. Given that this study presents one of the most extensive collections of the morphospecies *P.goniata* known to date and is the first to genetically characterize them, we suggest that *P.goniata* and *P.ios* are one species. As *P.goniata* is the younger name, we here synonymize it with *P.ios*, as per the International Code of Zoological Nomenclature Article 23.1. These species are thus treated as synonymous hereafter.

### ﻿Biogeography

*Provanna* are currently found in nearly every oceanic basin (Table 4). While many original descriptions distinguish species based on oceanic basin, the effect of geographic distance on population divergence of these gastropods from chemosynthetic habitats remains unclear (Distel et al. 2000; Vrijenhoek 2010; Breusing et al. 2023). For instance, while *P.laevis* (inclusive of *P.glabra*) spans the entire perimeter of the Northern Pacific Gyre with little genetic distinction, *P.laevis*, *P.kuroshimensis*, *P.lucida*, and *P.subglabra* all have overlapping biogeographic ranges at the Okinawa Trough yet display marked genetic divergence. *Provanna* are also notably adaptable across habitats, with six species currently known from more than one chemosynthesis-based ecosystem. These results indicate that more work is still needed to understand the drivers of genetic variation and isolation within this genus across a variety of contexts.

Finally, this study amends the biogeographic distribution of *P.muricata*. This species is listed as present in the North Fiji and Lau Basins in several secondary sources (Sasaki et al. 2016; Linse et al. 2019) based on Desbruyeres et al. (2006). This resource, however, does not present new records of occurrence, and instead summarizes known occurrence records. However, no primary literature nor museum specimens exist that place this species there. Therefore, until specimens are collected from the Western Pacific Basins and positively identified as *P.muricata*, this study proposes an amendment to their published biogeographic range, limiting it to the Eastern Pacific vents from which they were first found and described (Table 4).

## ﻿Conclusions

This study expands the ranges of *P.laevis*, *P.ios*, and *P.pacifica* to hydrocarbon seeps at the Pacific Costa Rica Margin. We also present a thorough review of the genus *Provanna*, consolidating the geographic distributions, genetics, and morphology for each extant species. We find that shell and radular morphological characters may be used to identify *Provanna* species and present the first identification key for this group. We also find that current species delineations within the genus *Provanna* are, for the most part, well-supported by genetic data. For those that are not, we herein synonymize *P.glabra* with *P.laevis* and *P.goniata* with *P.ios*. Future work will no doubt reveal new morphological varieties, species, and occurrences of *Provanna* snails. This key is designed to be a starting point from which researchers may begin this vital work.

## Supplementary Material

XML Treatment for Provanna laevis

XML Treatment for Provanna ios

XML Treatment for Provanna pacifica

## ﻿Additional information

### Author ORCIDs

Melissa J. Betters https://orcid.org/0000-0002-8975-257X

Erik E. Cordes https://orcid.org/0000-0002-6989-2348

### Data availability

Gene sequences generated in this study are accessible on GenBank under the accession numbers OM914402–OM914408, OP577954, and OR687645–OR687650. Morphological and genetic data from novel specimens are available on Github (Repository: melissajbetters/CRM_Provanna) or through correspondence with the lead author. The data underpinning the analysis reported in this paper are deposited at GBIF, the Global Biodiversity Information Facility, and are available at [https://doi.org/10.15468/4w9oc7] and [https://ipt.pensoft.net/resource?r=crm_provanna].
